# Classical and Modern Models for Biofilm Studies: A Comprehensive Review

**DOI:** 10.3390/antibiotics13121228

**Published:** 2024-12-18

**Authors:** Zhihe Yang, Sadaf Aiman Khan, Laurence J. Walsh, Zyta M. Ziora, Chaminda Jayampath Seneviratne

**Affiliations:** 1 School of Chemistry and Molecular Biosciences, University of Queensland, St. Lucia, QLD 4072, Australia; zhihe.yang@uq.net.au; 2 Oral Health Centre, School of Dentistry, University of Queensland, Herston, QLD 4006, Australia; sadaf.khan@imb.uq.edu.au (S.A.K.); l.walsh@uq.edu.au (L.J.W.); 3 Institute for Molecular Bioscience, University of Queensland, St. Lucia, QLD 4072, Australia; z.ziora@uq.edu.au; 4 Indian Institute of Technology (IITD) Delhi, University of Queensland, New Delhi 110016, India

**Keywords:** biofilms, laboratory models, micro-environments

## Abstract

Biofilms are structured microbial communities that adhere to various abiotic and biotic surfaces, where organisms are encased in an exo-polysaccharide matrix. Organisms within biofilms use various mechanisms that help them resist external challenges, such as antibiotics, rendering them more resistant to drugs. Therefore, researchers have attempted to develop suitable laboratory models to study the physical features of biofilms, their resistance mechanisms against antimicrobial agents, and their gene and protein expression profiles. However, current laboratory models suffer from various limitations. In this comprehensive review, we have summarized the various designs that have been used for laboratory biofilm models, presenting their strengths and limitations. Additionally, we have provided insight into improving these models to more closely simulate real-life scenarios, using newly developed techniques in additive manufacturing, synthetic biology, and bioengineering.

## 1. Introduction

The field of biofilm research was pioneered by William “Bill” Costerton, who initially studied marine biofilms [1]. In 1978, he defined the “biofilm” as “a bacterial aggregation adhered onto a surface” [2]. Today, biofilms are recognized as formed by various microorganisms, including bacteria and fungi [3]. A peculiar feature of microbial biofilms is the formation of an extracellular matrix from polymeric substances (EPS). This matrix can contain proteins, polysaccharides, and extracellular DNA [4]. The EPS plays a very important role, as it provides structural stability to the microbial community and protects inhabitants from external challenges [5]. Another key feature of biofilms is the presence of a highly resistant sub-population of cells, known as “persisters” (typically dormant cells that are highly tolerant to antibiotics and other stressors), that contribute to resistance to external agents [6]. 

The human gastrointestinal tract is colonized by a myriad of microorganisms. In health, these organisms maintain a symbiotic relationship with the host, but under certain circumstances, microbial biofilms on the surface of different parts of the gut can cause serious health issues [7]. Notable examples include ulcerative colitis and Crohn’s disease [8,9]. In the mouth, oral biofilms are responsible for the two most common chronic oral conditions, namely dental caries and periodontitis. Oral biofilms are formed on hard non-shedding surfaces (such as teeth, dental implants, prostheses, and appliances) as well as on soft tissues, such as the gingival tissues and the dorsal surface of the tongue [10]. Biofilms around teeth and dental implants are responsible for periodontitis and peri-implantitis, respectively [11]. 

Biofilms may cause infections in other parts of the body, such as in the urinary tract infections [12], which are common in hospitalized patients [13,14,15,16], especially when catheters and other medical devices are inserted or implanted [17]. In such situations, biofilms can grow on inert, nonliving (abiotic) material surfaces of the medical device. For instance, rougher surfaces are often more conducive to biofilm formation, and certain materials (e.g., silicone, polyvinyl chloride) may promote stronger microbial adhesion. Microorganisms can form stable permanent attachments on the medical devices and then synthesize the EPS, making them difficult to eradicate using antibiotics [18,19]. It is noteworthy that the material composition and surface topography of medical devices influence the adhesion of microorganisms and the formation of biofilms [20].

These clinical scenarios highlight the need to have suitable laboratory biofilm models that can simulate real-life situations. There are various factors that need to be taken into consideration when developing a suitable biofilm model, including the surface properties and chemical composition, the nature of the microorganisms, the presence of physical factors (such as shear forces created by fluid motion or agitation), and the growth conditions (temperature, redox potential, pH, nutrient levels, and others) [21]. The present review discusses the currently available and emerging laboratory biofilm models that could be used to test approaches to prevent biofilm formation and to eliminate existing biofilms, and how these model systems compare to one another. Our considerations are the (i) factors related to microorganisms; (ii) factors related to the substrate surface; and (iii) environmental factors that may modulate bacteria–surface interactions. The criteria for evaluating different approaches include reproducibility, scalability, and relevance to clinical settings, which were used to provide comparative information in this review.

## 2. Factors Influencing Biofilm Formation in Laboratory Models

### 2.1. Factors Related to Bacteria 

Based on the cell wall structure, the net electrical charge on bacterial cells can vary, which in turn affects electrostatic interactions with surfaces, nutrients, and other bacteria. The surface charge can be expressed as the zeta potential (electrokinetic potential). Due to the presence of lipopolysaccharides, the negative charge of Gram-negative bacteria can be many times greater than that of Gram-positive bacteria [22]. Altering the surface charge influences the initial adhesion and growth of bacteria [23]; a surface that is negatively charged is less suitable for the adherence of bacteria [24]. 

Bacterial appendages, such as fimbriae, pili, and flagella, help attach bacteria to surfaces [25], as well as to human cells [26]. Adhesin molecules on the fimbriae can support biofilm formation by promoting cell–cell contact between bacteria [27]. On the other hand, during the dispersal of bacteria from biofilms, flagella propel bacteria away from a biofilm, so that they can seed in other sites and form new colonies, thus perpetuating the biofilm circle of life [28].

### 2.2. Factors Related to the Substrate Surface 

**Figure 1:**
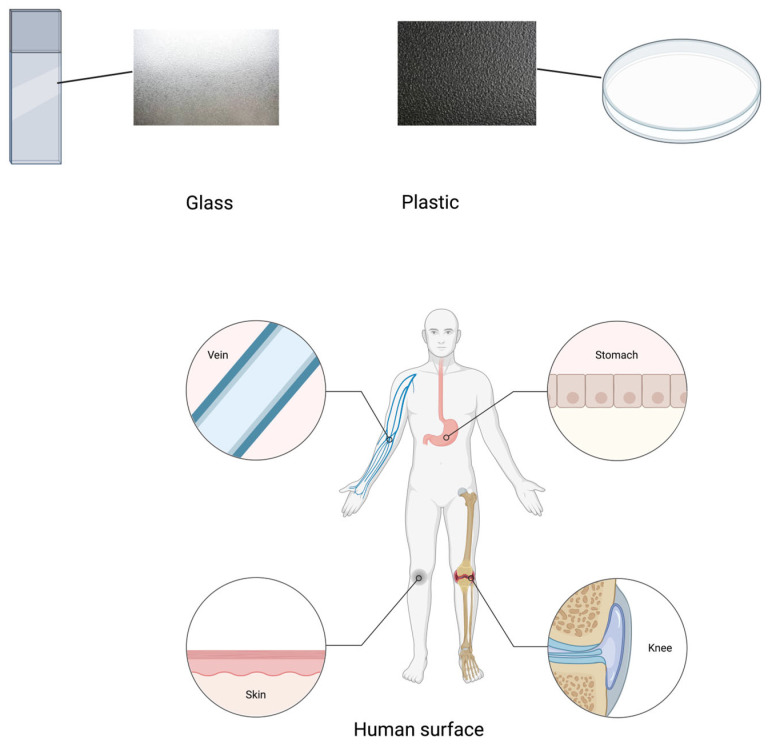
Human tissue surfaces (veins, stomach, skin, and knees) and artificial substrate surfaces (glass and plastic). Image made using 2024© Biorender.

Substrates used in laboratory biofilm studies to mimic intact tissue (in organ cultures or as organoids, or as cell monolayers in cultures) and those used in medical devices, such as metal alloys, glass, and various plastic polymers, differ in their texture, shape, and smoothness of the surface and their surface energy (Figure 1).

Silica and silicate glasses can be made with different textures and varying degrees of roughness. When in water, these glasses have a negatively charged surface, as terminal silanol groups dissociate [29]. Most plastic polymers have smooth surfaces and a neutral surface charge when formed by injection molding, but this charge can change to negative or positive with temperature as the material cools. Many common polymers such as acrylics, epoxies, polyethylene, polystyrene, polyvinylchloride, polytetrafluorethylene, polyesters, and polyurethanes are hydrophobic and will repel water [30]. Hence, the surface properties of the material used for the biofilm model can influence the way that water interacts with any biofilms that form on the surface. 

On the other hand, human tissue surfaces are microscopically irregular because of the surface cells and features such as villi, rugae, ducts, and pores. Mucosal surfaces are coated with a hydrated mucus, which is a complex mixture of water, proteins, and mucin glycoproteins. Mucus binds strongly to both neutral and charged surfaces (positive and negative) and attaches firmly to the hydrophilic outer layer of human cells. The distribution of charge within the various layers of mucus influences the binding of microorganisms, as does the removal of the mucus layer, which leaves the underlying hydrophilic cell surface exposed [31]. When the intact epithelial surface is wounded, the breach provides an opportunity for microorganisms to bind to host components exposed on the wound and evade host innate defenses such as the normally low-pH natural defense molecules [32]. Hence, rough and damaged epithelial surfaces are more conducive to the growth of biofilms such as those seen in diabetic foot ulcers. Table 1 summarizes key aspects of abiotic and biotic substrate surfaces that influence biofilm formation.

### 2.3. Factors Related to the Environment

Environmental factors that could impact the development of biofilms include temperature, pH, oxygen level, and the availability of nutrients [41]. The influence of temperatures in the range from 10 to 30 °C on biofilm growth has been studied using biofilm reactors [42]. Higher temperatures drive faster growth and metabolism, with enhanced oxidation of ammonia to nitrite [42]. For aciduric microorganisms, a lower pH (e.g., pH 5) enhances biofilm formation [43] and boosts EPS production [44]. Oxygen tension alters growth patterns of microorganisms depending on their nature, that is, whether they are obligate aerobic, facultative anaerobic, obligate anaerobic, aerotolerant anaerobic, or microaerophilic [45]. For aerobic organisms, the availability of oxygen favors biofilm growth. The level of available nutrients dramatically influences biofilm growth too. As an example, a deficiency in nitrogen sources can reduce biofilm biomass because nitrogen is the essential element for biofilm sustenance [46], and a surplus can accelerate biofilm growth.

## 3. Models for Biofilm Characterization

Biofilm models can be divided into three types: static models, flow-cell models, and modern models.

### 3.1. Static Model

A commonly used static model is the 96-well microtiter plate, either with or without agitation. In this method, preprepared planktonic cultures with the desired concentration of seeding bacteria are added to the microtiter plate, and adhesion to the polystyrene plastic surface occurs during the incubation period, leading to biofilm formation. Rinsing the plate at the end of the incubation period removes non-adherent microorganisms, leaving a surface-attached mature biofilm community for subsequent experiments. There are various techniques available to ascertain the biofilm biomass, including the commonly used crystal violet staining. This technique is based on staining bacterial cells [47] and polysaccharides present in the extracellular matrix [48] by triphenylmethane dye bound through ionic interactions. Crystal violet staining reflects the total biomass without separating viable cells from matrix components. Therefore, if needed, viable cells can be evaluated by plating the organisms on appropriate agar plates and counting the colonies, although this method is labor-intensive [49].

### 3.2. Flow-Cell Model

The flow-cell model is made of polystyrene that has been placed on a microscope slide and tubed to an outlet waste container and an intake medium vessel. An upstream position for a multichannel peristaltic pump is typically utilized to facilitate the flow of liquid medium [50]. The flow-cell concept is employed in multiple devices including chemostats, drip flow reactors, rotating biofilm reactors, constant-depth film fermenters, and the modified Robbins device [51]. 

#### 3.2.1. Calgary Device Biofilm Model (CBD) 

Traditionally, microtiter plate-based assays are used to determine the minimum inhibitory concentration (MIC) of antimicrobial agents against biofilm and its planktonic cultures. The Calgary biofilm device (CBD) provides a superior platform for MIC studies for testing agents used against biofilms. Biofilm forms on the pegs of the device. This approach has been used to test the susceptibility of biofilms of *P. aeruginosa* and *E. coli*, to antibiotics such as vancomycin and penicillin in terms of biofilm elimination concentration (MBEC), scanning electron microscopy (SEM), and other downstream experiments [52]. 

#### 3.2.2. Classic Flow-Cell Biofilm Model

An important variation on 96-well plates is the Calgary biofilm device, which has been used to assess susceptibility to antibiotics against bacterial biofilms. The Calgary biofilm device features a special lid with 96 pegs that can be removed without the risk of contaminating the biofilms. There is a consistent flow of growth medium across the surface of the pegs. The constant shear force ensures that the biofilms formed on each peg are equivalent.

**Figure 2:**
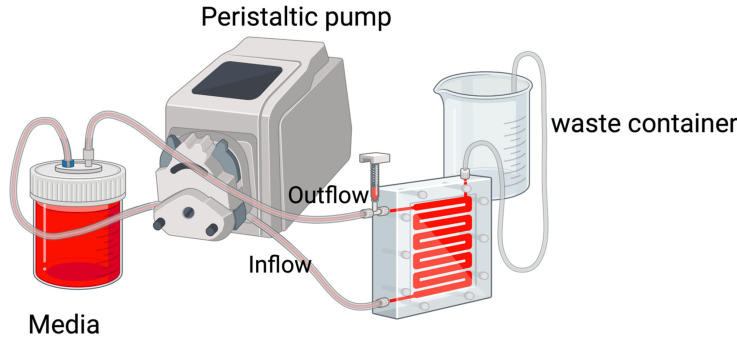
An example of the classic flow-cell model as used in Neiland’s research [53]. Image made using 2024© Biorender.

A classic flow-cell biofilm model is useful for observing biofilm growth and behavior. Biofilms are formed under dynamic flow conditions in this model, which has been popular for engineering and medical applications [51]. The system comprises two media and waste vessels and a flow cell, which is connected to a peristaltic pump (Figure 2). The media vessel provides the media and planktonic microbial solution to the system. The flow-cell technique allows for biofilm observation by microscopy as well as quantification. A slide is included in each flow cell, containing suitable substrata that allow biofilms to form within small channels under a constant flow of media. After passing through the flow cell, spent media are collected in a waste container [50].

The flow-cell biofilm model has been used to observe the formation of mono-species as well as mixed-species biofilms. The bacterial inoculum is placed in the media vessel and pumped through the flow cell, which allows biofilms to form on glass slides. The flow cell has a total volume of 2.1 mL and a surface area of 26 cm^2^. Analysis is based on the absorbance of the biofilm on the slides, as measured by a spectrophotometer [53].

#### 3.2.3. Chemostat 

**Figure 3:**
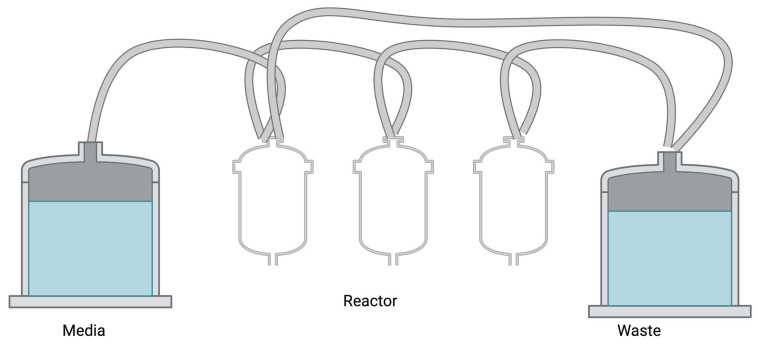
An example of a chemostat with three linked bioreactors. Image made using 2024© Biorender.

A chemostat is a continuously stirred bioreactor vessel where pH, temperature, oxygen level, nutrient levels, and flow rates are controlled to optimize biofilm formation. Fresh medium is continuously added, and pH is controlled through the addition of a buffer. Both the fresh medium and the buffer are supplied via their own peristaltic pumps. Oxygen is introduced at the bottom of the reactor chamber, and excess gas is vented from the top of the chamber. A regular outflow of waste medium is achieved using a peristaltic pump, to maintain a constant culture volume (Figure 3) [54].

#### 3.2.4. Drip Flow Reactor (DFR) 

**Figure 4:**
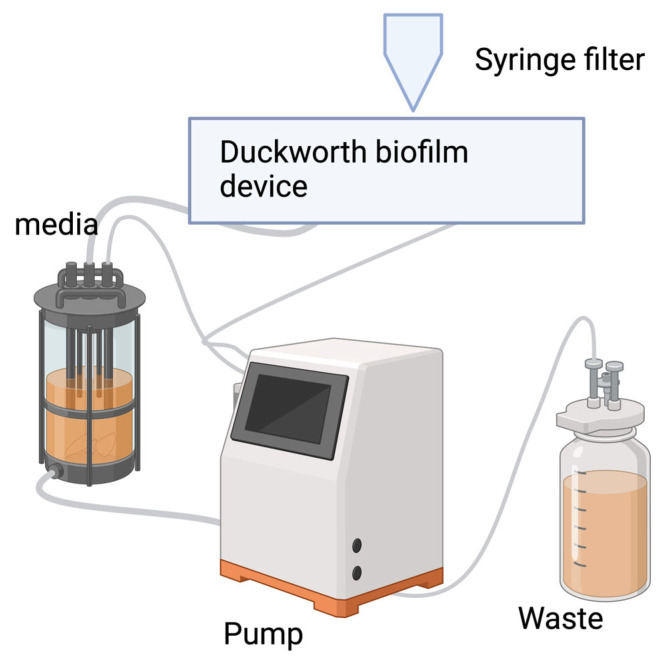
Connections within the model for a Duckworth biofilm device. Image made using 2024© Biorender.

The drip flow reactor has been used to model various biofilm-forming scenarios in a flowing medium. This reactor consists of multiple testing channels with glass slide coupons or other substrates for biofilm growth. Flowing medium passes drop by drop through the upper edge of the coupon and flows over the coupon. In this bioreactor system, fresh medium containing planktonic microorganisms is continuously introduced from the upper media vessel to the bioreactor via tubing and a peristaltic pump. After passing through the bioreactor, the waste medium is collected in the lower waste reservoir (Figure 4). There are several advantages of this system. For example, biofilms can be specially grown in the drip-flow mode and microscopic observation of the biofilms on the coupon can be conducted using a microscope. The biofilm biomass can be measured by harvesting the biofilm for further testing [55].

#### 3.2.5. Rotating Biofilm Reactor

The rotating biofilm reactor is used as a microbial photobioreactor. It consists of a glass vessel with a rotating disk. The lid has an inoculum port and multiple ports for media and oxygen inflow and outflow. The spent media and waste vessels are linked to the bioreactor via peristaltic pumps. The rotating biofilm reactor model is commonly employed in studying environmental wastewater biofilms [56]. 

#### 3.2.6. Constant-Depth Film Fermenter (CDFF)

**Figure 5:**
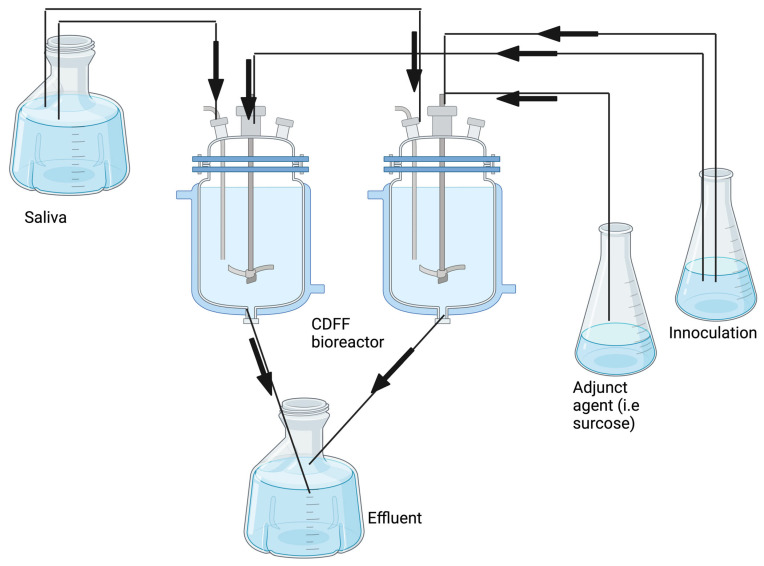
An example of a CDFF biofilm model. Each CDFF is loaded with twenty 4–8 mm hydroxyapatite (HA) disks. The *Streptococcus* spp. bacteria are grown at 37 °C for 72 h under anaerobic conditions (80% N_2_, 10% CO_2_, 10% H_2_). Image made using 2024© Biorender.

The constant-depth film fermenter is a steady-state biofilm model that has been used for studying dental plaque biofilm development and perturbation. It consists of a glass vessel with a steel plate. The top plate has ports for media and gas inflow while the bottom plate has medium and gas outflow ports (Figure 5). The reactor includes a stainless-steel disk containing fifteen polytetrafluoroethylene (PTFE) sample pans. The pans can rotate along a PTFE scraper bar, creating the maximum space for biofilm growth. In the constant-depth film fermenter system, coupons are suspended from the lid via a suitable holder. Fresh medium is pumped through the reactor using tubing and a peristaltic pump and biofilms form on the surface of the coupons [57].

#### 3.2.7. Modified Robbins Device (MRD) 

The MRD is an inline flow-type reactor used to study biofouling in industrial pipelines. The MRD model has been adapted to investigate biofilm growth in different environmental habitats, including those relevant to clinical settings. It is equipped with an inner sampling port surface for analysis, allowing multiple samples to be taken simultaneously at a single time point for biofilm growth. The biofilms grown on coupons can be removed for downstream analyses, such as staining and microscopy. One of the drawbacks of this model is that biofilms can be disrupted while removing the coupons [58]. To avoid disruption to the biofilms, in situ visualization can be achieved using real-time image analysis [59]. An MRD flow-cell system includes sterile media and planktonic media vessels, peristaltic pumps, a waste vessel, and the MRD reactor. The media and planktonic samples are pumped in as desired, similar to other flow-cell models [60].

### 3.3. Modern Biofilm Models

Modern biofilm models incorporate recent technical advancements and are used to better understand the complex nature of biofilm in real-life scenarios. The two major types of modern biofilm models are microfluidic models and impedance-based models.

#### 3.3.1. Microfluidic-Based Biofilm Models

**Figure 6:**
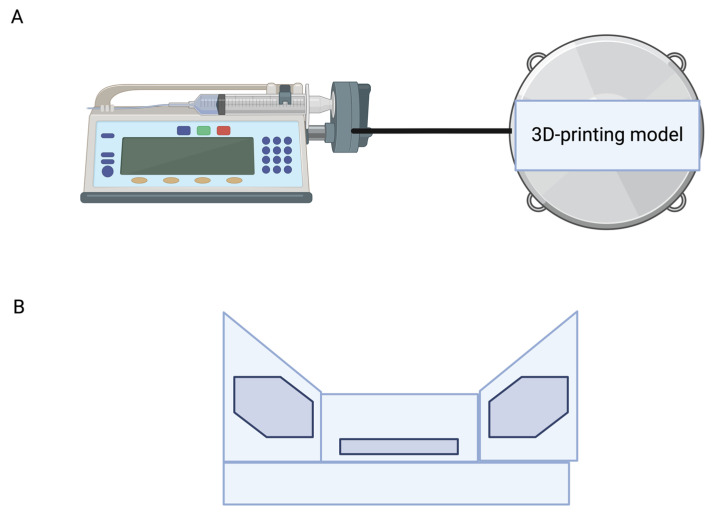
An example of a 3D-printed microfluidic chamber (**A**). The one-piece disposable model has an inlet, an outlet, and a central chamber with an open bottom for inserting the growth sample (**B**). Image made using 2024© Biorender.

Microfluidics are technologies used for handling and controlling liquids on a microscopic scale. The microfluidic biofilm model includes a microfluidic chip that is manufactured by soft lithography with polydimethylsiloxane. This silicone polymer allows for reproduction of the channels with micrometric features. The channels connect through inlets and outlets. When the system operates, the medium is pumped through channels allowing microbiofilm formation in the microfluidic chamber (Figure 6). The waste is then collected through an outlet channel on the opposite side of the chamber [61]. Compared to other flow chamber and flow system models, the microfluidic biofilm model offers more precise control of the hydrodynamic and physicochemical environment and better integration with analytical techniques. Hence, appropriately designed microfluid biofilm models can significantly contribute to our understanding of biofilms [61]. 

#### 3.3.2. Impedance-Based Technology

**Figure 7:**
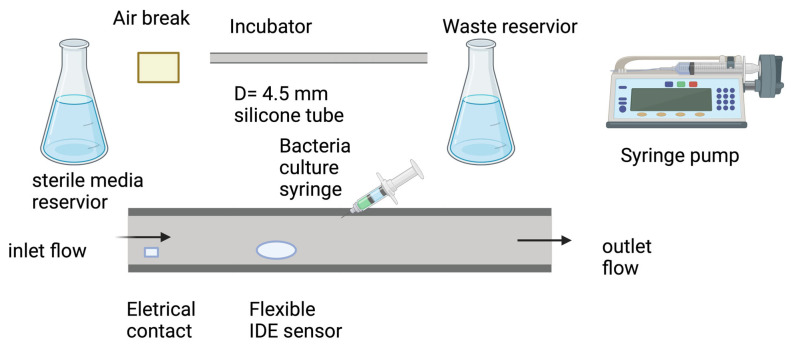
An example of a flow system setup for testing biofilms formed in a catheter. Image made using 2024© Biorender.

Impedance-based technology is a novel approach to monitoring the production of biofilm in real-time. In addition, pH sensors can be inserted into the model to observe the metabolic activity of the bacteria growing in biofilms on different biomaterials. For example, pH sensors can evaluate acid production by bacteria (Figure 7). The impedance-based method allows for real-time monitoring of the growth and metabolic activity of the biofilm, enabling better insights into the opportunities for developing strategies to control biofilms in clinical settings. 

The impedance-based biofilm model is connected to a computer to monitor the outputs from electrodes and pH sensors that are embedded within the biofilm [59,62].

## 4. Current Real-Life Models for Laboratory Biofilm Studies 

Researchers have attempted to develop clinically relevant laboratory models to study various real-life applications. Commonly used real-life laboratory biofilm models include environments such as the oral cavity, chronic wounds, medical implants, and device-associated biofilm models. 

### 4.1. Laboratory Biofilm Models Simulating the Oral Environment 

#### 4.1.1. Chemostat Flow-Cell Model Mimicking Real-Life Examples

Chemostat flow-cell models have been used to investigate the anti-caries properties of calcium glycerophosphate (CaGP). This model includes one chemostat reactor, four flow-cell techniques, and three solution vessels (CaGP, sucrose, and medium). The bacterial consortium (*S. gordonii*, *S. mutans*, and another five bacteria from dental plaque) is grown in a 250 mL chemostat bioreactor with 5% carbon dioxide and 95% nitrogen. The chemostat bioreactor pH is maintained at 7 by adding NaOH. The flow-cell model uses tooth sections and hydroxyapatite disks (to mimic tooth substance), with a mixture of media and the inoculum at a 9:1 ratio introduced at a constant 15 mL/h through the system. After the biofilm growth, fluid can be sampled to measure pH and assess bacterial populations [63].

#### 4.1.2. Constant-Depth Film Fermenter (CDFF) Biofilm Model 

A constant-depth film fermenter biofilm model has been used to investigate oral biofilm growth. This model comprises an inoculation vessel, a nutrient source (sucrose), a port for saliva on the top, CDFF, and a waste container. Silicone tubing connects the components, and a peristaltic pump produces continuous flow. CDFF devices can be loaded with hydroxyapatite (HA) disks as a substrate for biofilm growth. Biofilms form at 37 °C after 72 h under anaerobic conditions. Bacterial counts can be assessed as total colony forming units (CFU) present per mm^2^ [64].

#### 4.1.3. Microfluidic Flow-Cell Biofilm Model 

**Figure 8:**
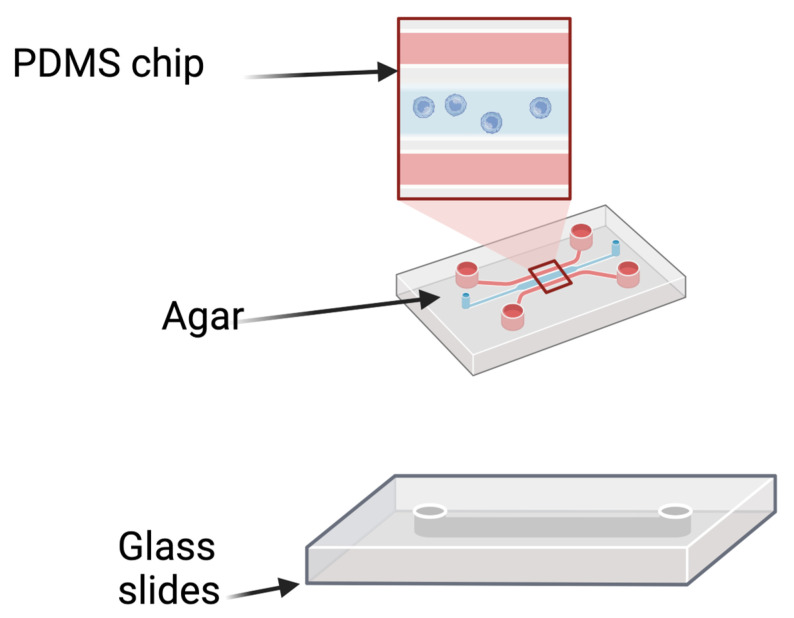
The components of a channel cell made using silicone. The syringe tube is used for the constant flow rate. A camera is used to capture activity in the main channel. Image made using 2024© Biorender.

In some oral cavity biofilm research, a 3D-printed microfluidic flow-cell biofilm model is used to analyze biofilm growth in situ (Figure 8). This flow cell includes an inlet, an outlet, and one 3D-printed chamber in which to place the growth sample. During the assembly, one cover slide is placed within the chamber for testing. Biofilms grow on the slides in the 3D-printed chamber to match the thickness of the saliva film in the oral cavity. The inlet is linked with a syringe pump and a reservoir filled with cleared saliva, sucrose, and media. An outlet tube is linked to a waste container. This model appears to be accurate in mimicking in situ conditions as it has a similar flow velocity and film thickness to the oral cavity [65].

Features of oral biofilm models are summarized in Table 2.

### 4.2. Chronic Wound Laboratory Biofilm Models

Researchers have explored various clinically relevant laboratory models for wound biofilms. Commonly used real-life laboratory biofilm models includes the Duckworth wound biofilm model and microfluidic chronic wound biofilm model. 

#### 4.2.1. Duckworth Wound Biofilm Model

The Duckworth wound biofilm flow system has been used to study biofilms related to chronic wound infection and to test the efficacy of antimicrobial wound dressings. Within the system, the bioreactor chamber is 3D printed from the resin, and it can be sterilized without affecting the accuracy of the device. During the assembly, the input is linked with the media and planktonic solution through the silicone tube and peristaltic pump. Then, the input flow is split into four channels before entering the reactor. Within the reactor, there are three wells with three agar disks in each channel. A syringe filter can be inserted above for pipetting each well with media or flowing the media in the well at 1 mL/min. In the end, the outflow connects to the peristaltic pump with a waste media container to collect the remaining solution. This system can be operated to obtain 48 h biofilms for downstream experiments. Samples are collected from the agar disks in the wells by forceps, which can be used for colony counting. Moreover, antimicrobial dressing against these in vitro wound biofilms can be tested by running the system for another 24 h and evaluating the biofilm population with the Accura ClearVue Resin device [69].

#### 4.2.2. Microfluidic Chronic Wound Biofilm Model

Wright et al. (2015) employed a microfluidic model combined with an imagining technique to study the behavior of *P. aeruginosa* in polymicrobial wound biofilms. The model system included the PDMS channel bioreactor, a syringe pump, and tubing. The PDMS channel is designed by CAD software (https://www.autodesk.com/solutions/cad-software accessed on 20 October 2024) and printed onto transparent film by the high-resolution image setter. The entire PDMS wound model was assembled with the glass slides as the base, followed by the agarose and PDMS chip layer. In this model system, the syringe pump is filled with the planktonic culture and media solution when in operation, which is then passed through the tube linked to the inlet of the microfluidic chamber. The tubing is removed when the solution fills the trench in the microfluidic chamber. The solution flows over the chip when the holes are sealed with PDMS. The microscopy camera integrated into the model captures the main channel’s cellular activity for further software analysis. Hence, this microfluidic chronic biofilm model allows for real-time observation of microbial activity in a complex wound biofilm environment, providing a better resolution to monitor the interactions between organisms [70].

### 4.3. Medical Implant Laboratory Biofilm Model

Rach et al. (2017) employed a flow-cell biofilm model to investigate biofilm formation on medical implants. In this model, the media vessel serves as the source of the bacterial culture connected to the bioreactor, which includes a glass coverslip to allow for microscopic analysis of biofilm formation on the surface. The solution is passed through the collector, peristaltic pump, and bubble trap, and then it is transferred to the flow-cell chamber. Afterward, the solution is collected in the initial bioreactor vessel for further circulation. The biofilms on implant surfaces can be imaged using various microscopy techniques. This system is useful in investigating the antimicrobial efficacy of innovative dental materials [71].

### 4.4. Chemostat Gut Model 

The chemostat bioreactor model is able to mimic the specific temperature of an organ or tissue environments, allowing for the examination of various biofilm-related diseases. Crowther et al. (2014) investigated the biofilm of Clostridium difficile infection (CDI) in the gut using this model. It included three chemostat bioreactors linked subsequently with waste containers and media to mimic the structure and connection mode of the gut. The first chemostat bioreactor is directly linked to the waste and media container, with another tube for comparison testing. Eighteen glass rods are inserted in each bioreactor, with an anaerobic atmosphere maintained by the delivery of nitrogen gas via a pipe. This model is proposed to mimic the gut epithelium. These “gut biofilms” can be scraped from the rod onto a plate for further bacterial population analysis [72].

### 4.5. Impedimetric Urinary Catheter Biofilm Model 

Similar real real-time monitoring laboratory biofilm models have been used in various other clinically relevant settings. Some research [73] used a laboratory flow-cell model to mimic interior urinary catheters. Similar to the aforementioned models, the medium in this system is run through the tubing to simulate a urinary catheter in real life. The biofilm growth is monitored via the impedance change data from the gold-interdigitated electrode in the catheter biofilm bioreactor. The media vessels are connected to the catheter tubing by a silicone tube and syringe pump to maintain the flow. Within the reactor, catheter tubing is coated with PDMS to help biofilm adherence. This platform represents a promising strategy to study urinary tract infections [73].

The representative models are summarized in Table 3.

## 5. Challenges and Limitations Associated with Laboratory Biofilm Models 

Although the classical and modern biofilm models highlighted in this work provide useful insights into the real-life scenarios, there are multiple limitations in the aforementioned models. Most of these models have only considered microbial and material aspects, without incorporating the host response to in vivo biofilms. It is conceivable that the host response is a major modulator of biofilm growth and behavior in health as well as disease. For example, dental plaque is an archetypical biofilm that constantly forms on the tooth surface in health. However, poor oral hygiene may lead dental plaque to accumulate, which can harbor pathogenic organisms, leading to diseases such as gingivitis and periodontitis. These diseases arise due to the inflammatory host response to the dysbiotic dental plaque biofilm. Therefore, in order to study the dental plaque biofilm, the host component should be included in laboratory models. However, such models are yet to be developed. This is also applicable to other biofilm models such as the gut biofilm model, catheter models, etc., mentioned in this review. 

Another challenge is the lack of an evaluation method for inter-species and host–microbial interactions in these models. Basic evaluation methods such as counting colony forming units, crystal violet assay, XTT assays, etc., are being used for most of biofilm studies. These methods are only able to provide a holistic view of biofilm growth and behavior, but not the complex interactions taking place within the biofilms [74]. Some researchers have attempted to develop species-specific probes to track the growth and interactions within the biofilms. This should be a future direction or research, as biofilm infections are often polymicrobial in nature. 

Moreover, in vitro biofilm models could be expanded to integrate novel smart material surfaces [75]. Physiochemical properties such as the flow rate, temperature, and pH control could be better incorporated into real-time biofilm monitoring systems. This would be very useful for pH-sensitive biofilm diseases such as dental caries. Another area that needs development is biofilms formed on surfaces subject to free-flow of fluids, such as dental unit waterline biofilms. Current models face significant limitations in testing the efficacy of disinfectants against polymicrobial biofilms [76]. The optimization of media that support a wide range of microorganisms formed on real waterline biofilm is itself a challenge, let alone those forming on multi-species biofilm on surfaces. Overall, the field of biofilms is in urgent need of real-life biofilm models using advanced technology, which will enable the incorporation of microbial, material, and host aspects in well controlled micro-environments. 

## 6. Conclusions

In conclusion, the present review provides an overview of commonly used classical and modern models to study biofilm formation, the factors influencing biofilm growth, and their limitations, along with future research directions. While the current models have advanced with real-time monitoring techniques, significant challenges remain in fully mimicking real-life conditions by incorporating microbial, material, and host aspects. Further research efforts are urgently needed to develop such real-life models, as biofilm is a major contributor to hospital-associated mortality and morbidity across a wide range of patients. 

## Figures and Tables

**Table 1:** Properties of different surfaces used for biofilm growth.

Substrate	Type	Example	Texture	Smoothness	Charge and Hydrophilicity	Surface Texture	Wettability (Surface Interaction with Liquids)
Glass [29]	Abiotic	Glass slides	Sleek	Smooth	Negative, hydrophilic	Regular	Evenly to form a film
Plastic [30]	Abiotic	Falcón tube	Soft	Smooth	Neutral (polyethylene), hydrophobic	Regular	Coating and fluid handling
Silicone [33]	Abiotic	Silicone tube (polydimethylsiloxane)	Soft	Smooth	Neutral	Regular	Attach
Metal alloys	Abiotic	Pure metal [34]	Laser	Smooth	Neutral (surface oxide), hydrophilic	Regular	Attach and expand
		Aluminum alloy [35]	Porous like	Smooth but partly rough		Regular	Attach and expand
		Steel [36]	Smooth and polished	Smooth		Regular	Attach and expand
		Dental alloy (titanium based) [37]	Soft	Smooth	Positive (pH < 6), negative (pH = 7–8), hydrophobic (silver with cysteine)	Irregular	Roll off
Skin	Biotic	Outer skin [38]	Smooth skin pores, fine lines, wrinkles	Smooth	Slightly positive (body) and negative (tissue) charges	Irregular	Water interacts (absorbs or holds moisture)
Mucosa	Biotic	Oral [31]	Epithelium, Papillae, Rugae, gland ducts	Smooth	Negative, hydrophobic	Irregular	Attach and retain moisture
	Biotic	Gut [39]	Furry	Smooth	Negative, hydrophobic	Irregular	Attach and interact with water
Tooth enamel	Biotic	Enamel in acid [40]	Etched	Rough	Neutral, hydrophobic	Irregular	Attach

**Table 2:** Features of laboratory models used in oral biofilm studies.

Model	Flow/Static	Analysis Device	Growth Surface	Microbes	Limitation and Strength	Comment
Chemostat [63]	Flow	pH meter	Glass	*S. gordonii*, *S. salivarius*, *S. mutans*, *A. naeslundii*, *V. parvula*, *F. nucleatum*, *P. nigrescens*	L: Difficult to build S: Mimics real health scenario (intestines, stomach)	Reduced enamel demineralization with increasing concentration of calcium glycerophosphosphate in this bacterial flow-cell model.
Flow cell [53]	Flow	Fluorescence spectroscopy	Glass slides	Saliva fraction	L: Cannot control temperature S: Easy to build (all sources can be found in normal lab)	Loss of biofilm homeostasis leads to dysbiosis, which drives the development of oral diseases such as dental caries and periodontitis.
Constant-depth film fermenter (CDFF) [64]	Flow	Plate count	Glass	Saliva fraction	L: Expensive, hard to build S: Controls PH, temperature, and wettability	This model can improve the sensitivity for experiments that assess the effects of antimicrobial agents in nutritional supplements.
Microtiter plate [66]	Static	Scanning electron microscopy (SEM), confocal laser scanning microscopy (CLSM)	Plastic (polystyrene)	*F. nucleatum*, *P. gingivalis*	L: Static model S: Easy to buy and build	Changing between dynamic and static methods did not influence biofilm thickness in this study.
Calgary device [67]	Static	Community and structure principal coordinate analysis	Plastic (polystyrene)	Saliva	L: Cannot control temperature S: Easy to build	Diverse oral biofilms can be grown and maintained using this device. It can also be used to test antimicrobial agents and to assess the influence of probiotic bacteria.
Microfluidics [53,65]	Flow	pH meter	Glass	Saliva fraction	L: Source (PDMS) difficult to obtain S: Small and convenient to bring	As stimulated flow of saliva occurred, the pH rose to neutral or slightly higher in all biofilms, and deeper parts of biofilms were more acidic than the surface parts.
Impedance-based technology [68]	Static	Fluorescence spectroscopy	Plastic	Saliva, Tongue, and supragingival plaque fractions	L: Need to build software S: Can obtain real-time data	This model can be used to test the influence of antibiotics, antiseptics, and anti-adhesive compounds.

**Table 3:** Features of laboratory biofilm models for medical devices.

Model (Reference)	Flow/Static	Analysis	Growth Surface	Microorganisms
Drip flow wound biofilm model [69]	Flow	MIC	Plastic	*P. aeruginosa*, *S. aureus*
Modified Robbin chronic wound biofilm model [70]	Flow	Fluorescence spectroscopy	Glass	*P. aeruginosa*
Chamber model of oral implant [71]	Flow	Fluorescence spectroscopy	Glass	*S. gordonii*, *S. oralis*, *S. salivarius*, *P. gingivalis*, *A. actinomycetemcomitans*
Chemostat gut model [72]	Flow	Biofilm biomass	Glass	*Clostridium difficile* and human microbiota
Urinary catheter biofilm impedance model [73]	Flow	Voltage	Plastic	*E. coli*

## Data Availability

No new data were created or analyzed in this study. Data sharing is not applicable to this article.
